# Effects of Opium Use on Cardiovascular Mortality: A Critical Appraisal of a Topic

**Published:** 2019-10

**Authors:** Seyyed Ali MOEZI, Nahid AZDAKI, Toba KAZEMI, Hamid Reza MASHREGHI MOGHADDAM, Neda PARTOVI, Fatemeh HAMIDI, Nazanin HANAFI-BOJD, Majid JAFARNEJAD, Samaneh NAKHAEE

**Affiliations:** 1. Cardiovascular Diseases Research Center, Department of Cardiology, School of Medicine, Birjand University of Medical Sciences, Birjand, Iran; 2. Medical Toxicology and Drug Abuse Research Center (MTDRC), Birjand University of Medical Sciences, Birjand, Iran; 3. Student Research Committee, Birjand University of Medical Sciences, Birjand, Iran

## Dear Editor-in-Chief

In many Asian and Middle Eastern countries such as Iran, the common use of opium as a treatment for cardiovascular problems has been discussed. Nevertheless, there are few studies to prove or disprove this belief (1). However, to the best of our knowledge, several studies have been carried out to address the effects of opium on cardiovascular disease and its risk factors but very low comprehensive article has been written regarding its effect on mortality of CVD, so our clinical question was raised in this specific area of care.

To answer this clinical question, the literature search was performed. A review of the abstracts of these articles led to the selection of one article (2) that assessed the effects of opium on overall and cause-specific mortality. This study is the largest cohort study in Iran and has the highest relevance to our clinical question and also the highest level of evidence, so we decided to appraise this topic critically (CAT it).

For critical appraisal of this topic, we selected a tool which is a critical appraisal tool relating to public health issues (3).

This study is a prospective cohort study with large sample size and prior validation of exposure and outcome measurements and the follow-up information of participants for over 99% was obtained. This study would have Level 2b evidence and strength of recommendation Grade B.

In this study, the cause of death in 35% of the cases was determined just based on verbal autopsy which can cause bias in the measurement. Moreover, the blinding of the person who performed statistical analysis on the exposure data was not mentioned. It can cause analytical bias.

### Additional notes

**Fig. 1:**
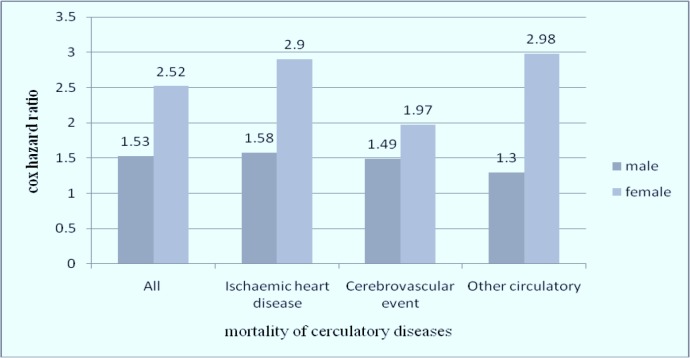
Hazard ratios for circulatory mortality in relation to opium consumption in males and females

▪ Risk of overall and cause-specific mortality, independent and non-dependent groups after four years of follow-up are shown in Table 1. The value for NNH of overall mortality is lesser than one. Twenty people consuming opium would lead to one additional patient being harmed, compared with patients who do not use opium. This value for Ischemic heart disease is 71.42. In overall, the higher the NNH, the less harmful is the exposure.
  According to the results of this study, the hazard ratios of cause-specific mortality were higher in women than in men (Fig.1) except for cancer of the stomach and lung, asthma, and liver cirrhosis although the reported confidence interval for these values is very large in some cases.
▪ In the current study, criteria for opium dependency were just based on participant's reports. The agreement between self-report of opium use and positive urinary sample was assessed in 150 participants. However, while opium is an illicit drug and there is a stigma associated with drug addiction in Iran, some people may hide their opium addiction which can cause bias in the measurement. Therefore, if the criteria for opium dependency such as DSM-IV criteria or urinary/blood test of morphine were considered for all participants, the accuracy of result could improve.
▪ The exposure information was assessed just at the beginning of study and it was not repeated in the follow-up period. In this time, some addicted people may quit their addiction or change their pattern of addiction or some people from non-exposed group may start opium use which can cause bias in the measurement.
▪ In this study, the exact time of home visits of the dead person for verbal autopsy were not clearly explained, the number of days after death the visits were performed were not defined. The closeness of this time to the death of participant can influence the response of family members.
▪ To the best of our knowledge, the effect of opium addiction with cigarette smoking and opium use alone on mortality or other cardiovascular consequences is not well documented. Therefore, additional researches are required to identify whether cigarettes have synergistic effects on these consequences or not.
▪ Moreover, the effects of opium abstinence on mortality or other cardiovascular consequences were not well documented in the literature, so the result of future studies in this line can help in choosing the best abstinence method in addicted patients.

**Table 1:** Risk of overall and cause-specific mortality in opium user and non-user groups after four years of follow-up

***Outcomes***	***Groups***	***Adverse outcome***		***Totals***	***Relative risk/hazard ratio***	***Patient's risks of adverse event***
		***Present***	***Absent***			
Overall mortality	Opium user	705	7782	8487	Rr=2.41	Ari=4.9%
					Hr=1.86	Nnh=20.4
	Non-opium user	1440	40118	41558		
	Totals	2145	47900	50045		
Circulatory diseases	Opium user	335	8152	8487	Rr=2.29	Ari=2.2%
	Non-opium user	738	40820	41558	Hr=1.81	Nnh=45.4
	Totals	1073	48972	50045		
Ischaemic heart disease	Opium user	208	8279	8487	Rr=2.4	Ari=1.4%
	Non-opium user	416	41142	41558	Hr=1.90	Nnh=71.42
	Totals	624	49421	50045		
Cerebrovascular event	Opium user	98	8389	8487	Rr=1.80	Ari=0.49%
	Non-opium user	254	41304	41558	Hr=1.68	Nnh=204.08
	Totals	352	49693	50045		
Other circulatory diseases	Opium user	29	8458	8487	Rr=3	Ari=0.2%
	Non-opium user	68	41490	41558	Hr=1.73	Nnh=500
	Totals	97	49948	50045		
